# Spatial Probability Dynamically Modulates Visual Target Detection in Chickens

**DOI:** 10.1371/journal.pone.0064136

**Published:** 2013-05-29

**Authors:** Devarajan Sridharan, Deepa L. Ramamurthy, Eric I. Knudsen

**Affiliations:** Department of Neurobiology, Stanford University School of Medicine, Stanford, California, United States of America; University of Sussex, United Kingdom

## Abstract

The natural world contains a rich and ever-changing landscape of sensory information. To survive, an organism must be able to flexibly and rapidly locate the most relevant sources of information at any time. Humans and non-human primates exploit regularities in the spatial distribution of relevant stimuli (targets) to improve detection at locations of high target probability. Is the ability to flexibly modify behavior based on visual experience unique to primates? Chickens (*Gallus domesticus*) were trained on a multiple alternative Go/NoGo task to detect a small, briefly-flashed dot (target) in each of the quadrants of the visual field. When targets were presented with equal probability (25%) in each quadrant, chickens exhibited a distinct advantage for detecting targets at lower, relative to upper, hemifield locations. Increasing the probability of presentation in the upper hemifield locations (to 80%) dramatically improved detection performance at these locations to be on par with lower hemifield performance. Finally, detection performance in the upper hemifield changed on a rapid timescale, improving with successive target detections, and declining with successive detections at the diagonally opposite location in the lower hemifield. These data indicate the action of a process that in chickens, as in primates, flexibly and dynamically modulates detection performance based on the spatial probabilities of sensory stimuli as well as on recent performance history.

## Introduction

The real world presents us with an abundance of sensory information. Much of this information is of little relevance, and may be ignored. On the other hand, sensory information that is relevant to an animal’s survival must be rapidly identified and acted-upon. The ability to flexibly and rapidly locate the most relevant information at any time is crucial for adaptive survival [1]. Relevant sensory information could be difficult to detect and is often limited in time and space. Anticipating the location of relevant sensory stimuli based on visual experience greatly enhances an animal’s ability to detect and respond to such stimuli, providing a significant adaptive advantage that manifests over various timescales, from very short (minutes) to very long (evolution).

Humans perceive most visual features with greater sensitivity, and higher resolution, when stimuli are located in the lower versus in the upper visual field [2], an asymmetry that confers a distinct lower visual field advantage in various tasks, such as locating a target among distractors [3] or in making fine discriminations about a target’s spatial features [4], [5]. In addition, they exhibit a systematic lower visual field advantage in the control of visually-guided actions, such as pointing and grasping [2]. These biases are hypothesized to result from differential selective pressures (over evolutionary time, or over an animal’s lifetime) for processing and acting upon stimuli close to the body in the lower visual field versus searching for approaching danger in the upper visual field [6]. In addition, human and non-human primates are able to exploit spatial regularities in the distribution of stimuli over short timescales (minutes), based on recent experience, to improve detection at locations of high stimulus probability [7]–[11]. Are these perceptual asymmetries and performance biases unique to primates, or do they operate in other species as well?

Here, we address this question in chickens. Chickens forage the ground for seeds and small insects. The likelihood of encountering a food item is far higher in the lower than in the upper visual field. The differential selective pressure hypothesis predicts, therefore, that chickens should exhibit an advantage for detecting small stimuli located in the lower versus the upper visual field. Does this prediction hold true, and does altering the spatial distribution of behaviorally relevant stimuli modulate this lower visual field advantage?

To answer these questions, we developed a multiple alternative detection (Go/NoGo) task for quantifying the ability of chickens to detect stimuli located in specific regions of the visual field. The paradigm, modeled on paradigms used in primate research, allowed us to measure location specific contrast-response functions based on response accuracy and reaction time, the same behavioral metrics that have been used to document perceptual visual field advantages in humans [10], [12]. With this paradigm, we show that, when the presentation of targets is not biased towards any one location in space, chickens exhibit a distinct lower visual field advantage in target detection, in line with the differential selective pressure hypothesis. Next, we show that increasing the probability of occurrence of targets at the upper field locations improves detection performance at these locations, thereby reducing the lower field advantage. Finally, we show that modulation of detection performance can be rapid and depends on the recent, trial-by-trial history of performance. The results indicate that processes that modulate detection and action based on the distribution of behaviorally relevant features at both short and long timescales are shared across vertebrate species.

## Results

### A Multiple Alternative Detection (Go/NoGo) Task for Chickens

**Figure 1:**
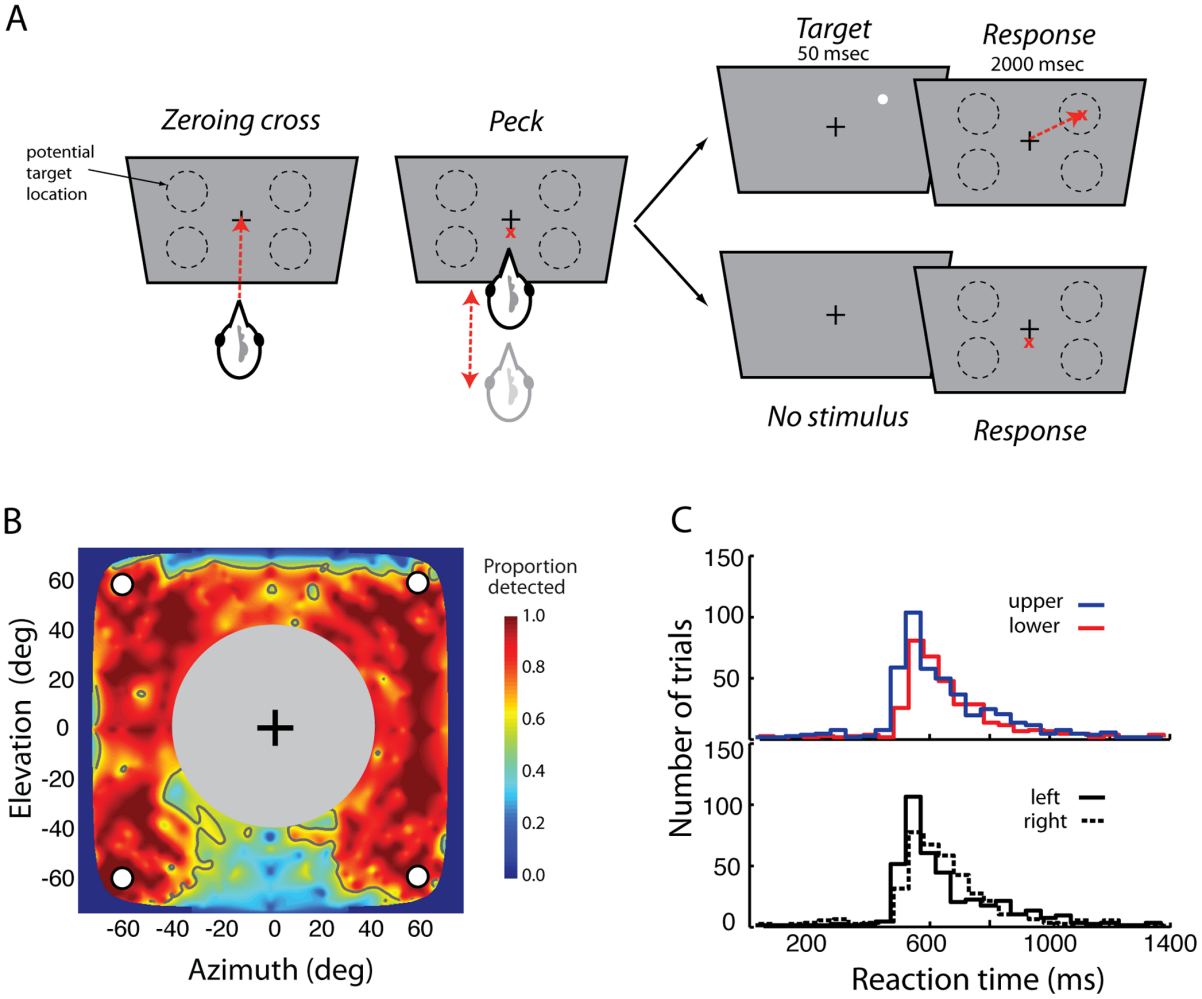
A multiple alternative (Go/NoGo) task for measuring visual detection performance at multiple locations. **A.** Behavioral paradigm. The chicken initiated a trial by pecking on the zeroing cross in the center of a touch-sensitive screen. Immediately following the peck, a 3° bright dot (target) was flashed for 50 ms at one of four specific locations on the screen, one in each quadrant in the bird’s visual field (Go trial, top panel). The chicken pecked at the location of the target, following its disappearance, to receive reward (upper panel). *(Bottom)* On interleaved NoGo trials, no target appeared following the peck on the zeroing cross, and the chicken pecked on the cross a second time to receive reward. **B.** Proportion of full-contrast targets successfully detected at various locations in the visual field (n = 9 experiments in 3 birds, 2674 trials). Hotter colors (reds) correspond to higher detection rates. Horizontal and vertical axes: Elevation and azimuth, in degrees of visual field (see Materials and Methods). Targets were presented at a fixed number of finely spaced locations across the visual field, and detection performance between these locations was interpolated (see Materials and Methods). Grey contours: threshold detection rate (50%). White circles: locations in the four visual quadrants where targets were presented in all subsequent experiments (±60° azimuth and ±60° elevation). Filled gray circle with black zeroing cross in the center corresponds to untested locations. **C.** *(Top)* Distribution of reaction times for detecting full-contrast targets presented at locations in the upper (blue) and lower (red) hemifields. *(Bottom)* Distribution of reaction times for detecting full-contrast targets presented at locations in the left (solid line) and right (dashed line) hemifields. Reaction times for detecting full contrast targets were not significantly different either between the upper and lower or left and right hemifields (p>0.05, Wilcoxon signed rank test).

Reliability and speed of detecting small stimuli of various contrasts were measured for 3 adult white leghorn chickens trained on a multiple alternative detection (Go/NoGo) task. The birds were trained to report the location of a 3° bright dot (“target”) on a touch-sensitive screen (Figure 1A). Trials were initiated when the bird pecked on a zeroing cross located at the center of the screen. This action oriented the bird’s head to a standard starting position, which was maintained for up to 250–300 ms following the peck on the cross (Figure S1), enabling us to present target stimuli in specific quadrants of the bird’s visual field. In “Go trials” (Figure 1A, top), immediately following the peck on the cross, a target was flashed for 50 ms, usually at one of four locations (see below). Birds pecked at the location of the target’s appearance to receive reward (hit trials). On interleaved “NoGo trials” (Figure 1A, bottom), no target was presented following the initial peck on the cross and the bird pecked a second time on the cross to receive reward (correct rejection trials). During training, NoGo responses on Go trials (misses) were not rewarded, whereas Go responses on NoGo trials (false alarms) were penalized (see Materials and Methods), so that the birds adopted a conservative strategy when reporting targets. Data collection began once each bird performed this task reliably with >90% correct Go responses to full contrast stimuli and nearly 100% correct NoGo responses.

In a preliminary experiment, we measured the birds’ ability to detect the target stimulus across a wide range of locations. This experiment permitted us to identify regions in the visual field where target detection was poor at baseline. These locations were then excluded in subsequent tests (see below). For this experiment, each bird was tested with only Go trials and full contrast targets, presented at random locations in a large zone (Figure 1B). The results are summarized in Figures 1B,C. For most of these locations, the birds reported the target on >97% of the trials (n = 9 experiments in 3 birds, 2674 trials). Only when targets were located in the upper and lower regions near the midline, corresponding to regions obscured by the forehead and beak, respectively, did the performance of the birds decline steeply (grey contour, Figure 1B). The sharp spatial definition of these regions, even after averaging across birds (Figure 1B), demonstrates the stereotypy of the position of the bird’s head relative to the screen when the targets were presented.

Reaction times were also similar across the high-performance locations in the visual field. Reaction time was measured as the time from the initial peck on the cross until the peck on the target location. Reaction times averaged 608±16 ms for the upper visual field and 630±14 ms (p = 0.17) for the lower (Figure 1C, above), and they averaged 624±14 ms for the left hemifield and 615±18 ms (p = 0.67, Wilcoxon signed rank test, n = 9 experiments) for the right (Figure 1C, below). These data indicated that the birds detected full contrast targets with similar reliability and speed across the locations tested in this study.

**Figure 2:**
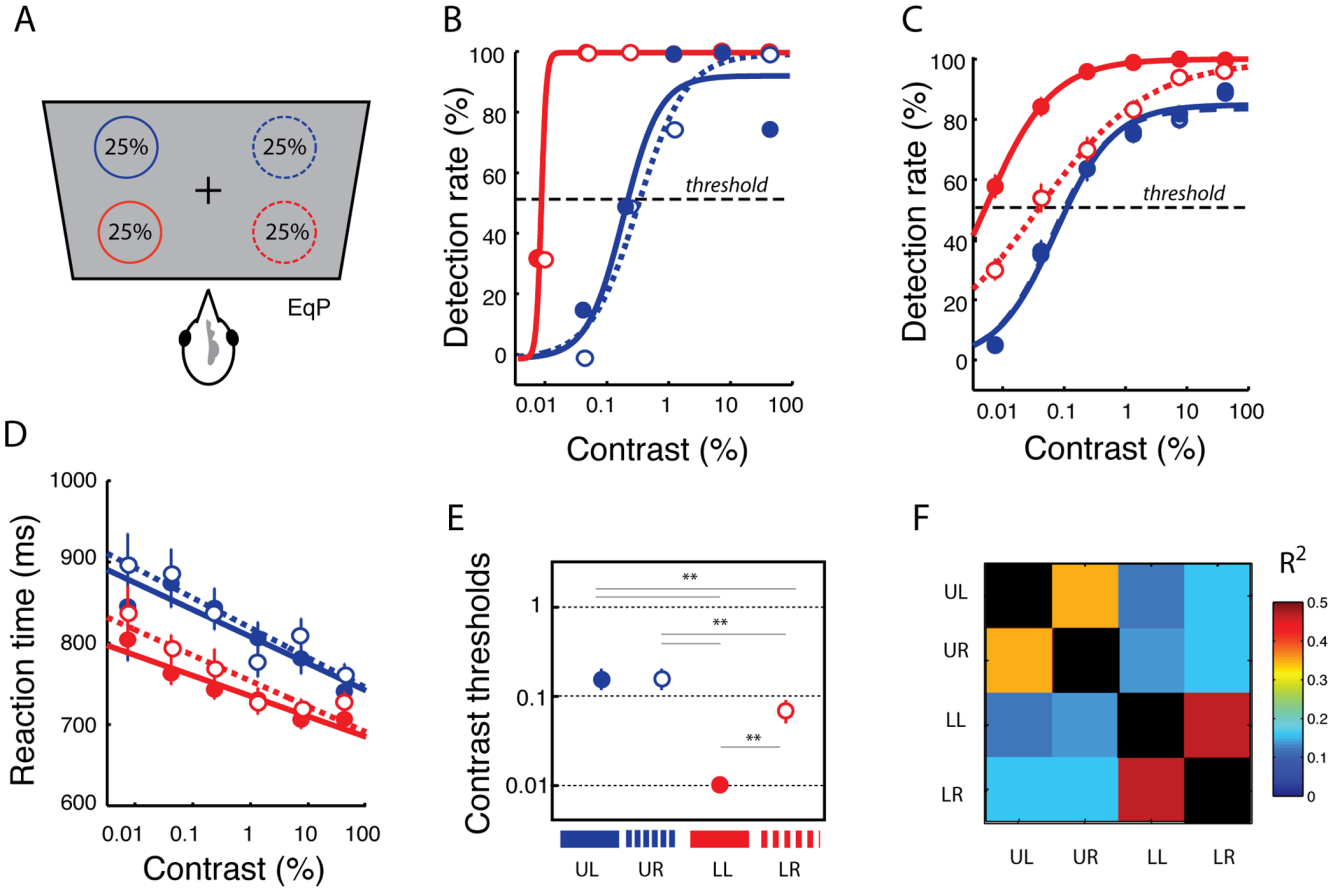
A lower visual field advantage for target detection. **A.** Targets were presented with equal probability (25%) in each of the four visual quadrant locations. **B.** Contrast-response functions showing the effect of target contrast on detection rates for a single bird (bird #2) measured within a single experimental session in the EqP condition. Target contrasts were log-transformed and binned at equal intervals. Circles: mean detection rates for target contrasts within each bin. Lines: sigmoidal fits. Blue circles: upper hemifield data. Red circles: lower hemifield data. Solid lines and filled circles: left hemifield data. Dotted lines and open circles: right hemifield data. Dashed horizontal line: threshold detection rate (50%). **C.** Summary data showing contrast-response functions of target contrast based on detection rates in the EqP condition (n = 88 experiments in 3 birds). Error bars: standard error of the mean (SEM) across experimental sessions. Other conventions are the same as in (B). **D.** Summary data showing mean reaction times as a function of target contrast in the EqP condition (n = 88 experiments in 3 birds). Blue circles: mean reaction times for targets in the upper visual quadrants. Red circles: mean reaction times for targets in the lower visual quadrants. Lines: linear fits. **E.** Mean contrast thresholds for the different visual quadrants in the EqP condition (n = 88 experiments in 3 birds). Circles: mean threshold values. These values, obtained by averaging contrast thresholds derived from sigmoidal fits to individual test session data, are similar to the contrast thresholds derived from sigmoidal fits to the mean data across test sessions, shown in (C). Error bars: standard error of the mean (SEM) across experimental sessions. Thin grey lines with asterisks: p<0.01 for the difference in contrast thresholds (ANOVA). Dashed horizontal lines indicate specific contrast values. **F.** Pair-wise correlation matrix (R^2^ values) of the detection thresholds across test sessions among the four visual quadrants. Warmer colors correspond to higher correlations (legend). Correlations along the diagonal are all unity (black).

For all subsequent experiments, detection performance was measured at four locations each centered in one of the visual field quadrants at ±60° azimuth and ±60° elevation (Figure 1B, white circles). In the following sections we present results for analyses performed by pooling data from experimental sessions across birds (except for Figure 2B, 3B). Analyses of data from individual birds are reported in the Supporting Information.

**Figure 3:**
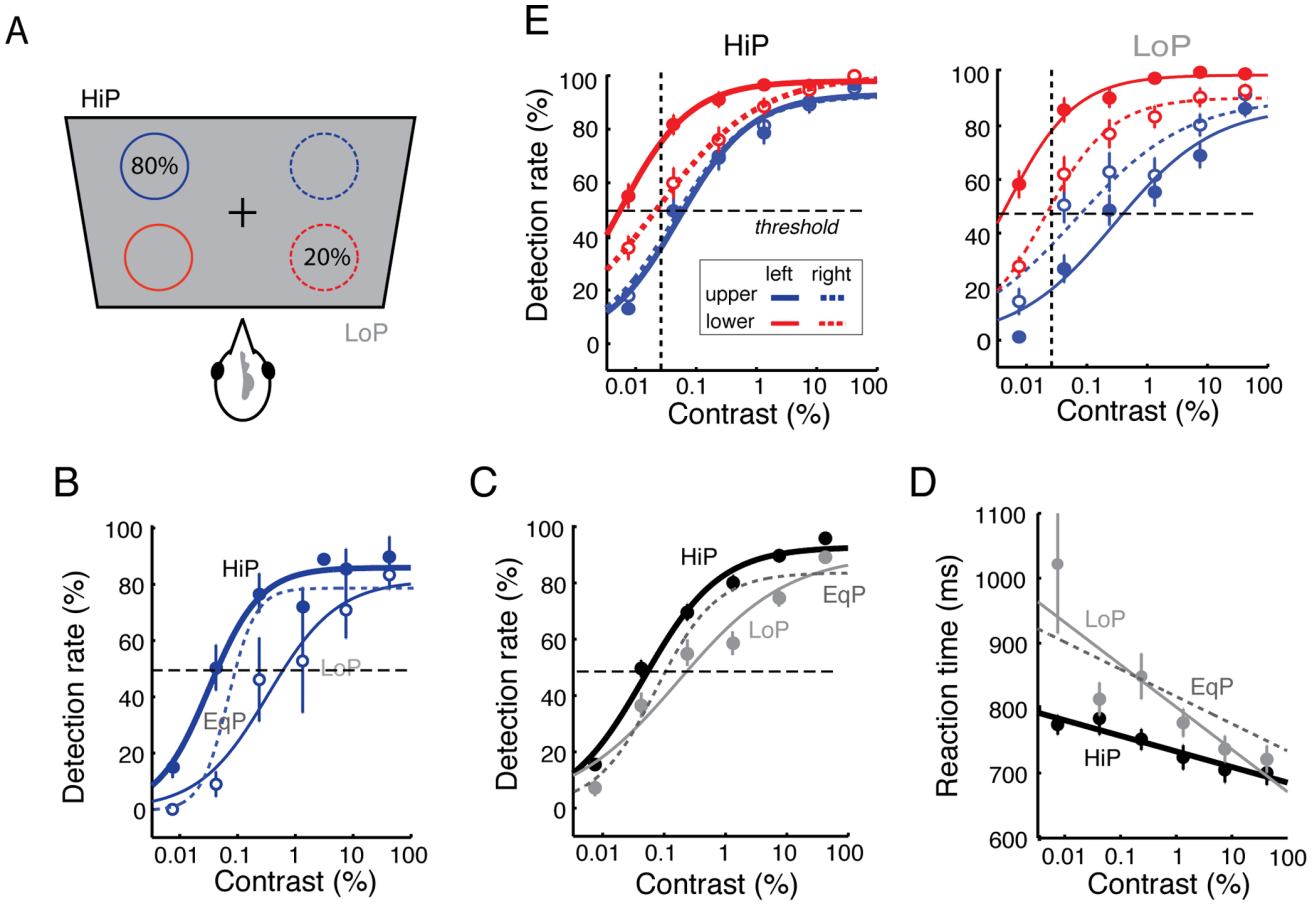
Higher target spatial probability improves detection performance in the upper hemifield. **A.** Stimulus configuration for modulating target spatial probability. In a test session, targets were presented with 80% probability at a location in one of the four quadrants (high-probability, HiP, black) and with 20% probability at the opposite location (low-probability, LoP, grey). In the next session, locations of HiP and LoP presentation were interchanged. **B.** Detection rates for upper hemifield targets from a representative bird. HiP detection rates: thick blue line; LoP detection rates: thin blue line. The contrast-response curve for the EqP condition (dotted line) from the same bird is shown for reference. **C.** Population summary data for detection rates of upper hemifield targets under each condition. HiP: black data; LoP: grey data. Lines: sigmoidal fits. The contrast-response curve for the EqP condition (dashed sigmoidal fit) is shown for reference. Other conventions are the same as in Figure 2C. **D.** Summary data showing mean reaction times as a function of target contrast in the HiP and LoP conditions. Lines: linear fits. Other conventions are same as in (C). **E.** *(Left)* Detection rates for targets in the four visual quadrants when the probability of presentation was high (80%, HiP) at that location. Dashed horizontal line: threshold detection rate (50%). Dashed vertical line: threshold detection contrast (0.025%). Other conventions are the same as in Figure 2C. (Right) Same as the left panel, except that detection rates were measured when the probability of presentation was low (20%, LoP) at that location. Contrast-response curves for the HiP condition were considerably more clustered compared to those for the LoP condition.

### Asymmetries in Detecting Targets at Upper Versus Lower Hemifield Locations

We measured the effect of changing target contrast on detection performance (“contrast-response functions”) at the four standard locations, one in each visual field quadrant (Figure 1B, white circles). Target location was varied in a pseudo-random order among the four locations with equal probability (Figure 2A, “EqP”; 25%), and the contrast of the target at each location was stair-cased independently toward threshold (Figure S2; Materials and Methods). We computed contrast-response functions based on percent detection and reaction times at each location, for each experimental session, and for each bird. Because guessing during NoGo trials was strongly discouraged during training, false alarm rates at all locations were negligible (median false alarm rates <2%). Thus, the percent of responses to a location was proportional to the bird’s percent detection (hit-rate) at that location.

Data from a single experimental session for one bird are shown in Figure 2B. The data are coded according to the visual field quadrant of the stimulus, as indicated in Figure 2A. For all quadrants, the bird was highly efficient at detecting targets when they were high contrast (>10%). However, as target contrast decreased below 1%, detection rates for targets in the two upper field quadrants declined sharply while detection rates in the two lower quadrants remained high (Figure 2B, blue versus red). Detection rates declined in the lower quadrants, only when the target’s contrast fell below 0.02%. Thresholds for stimulus detection were derived from these data. Threshold was defined as the target contrast that yielded a 50% detection rate, based on the sigmoidal fit to each data set (Figure 2B; Materials and Methods). Detection thresholds in this experimental session were an order of magnitude lower for targets in the lower (0.01%, Figure 2B) versus the upper (0.2% and 0.3%) visual quadrants.

Summary data across all test sessions from the population of birds (n = 88 experiments in 3 birds) were consistent with these results. Population average contrast-response functions for each quadrant are shown in Figure 2C. As a group, the birds exhibited superior detection rates when targets appeared in the lower versus the upper visual field locations. Contrast-response functions and detection thresholds were shifted toward lower contrasts (leftward) for stimuli in the lower versus the upper quadrants (Figure 2 C,E, p<0.01, one-way ANOVA followed by post hoc Tukey range test, n = 88 experiments pooled across birds). The contrast-response functions for the individual birds are shown in Figure S3.

Population average contrast-response functions based on reaction times showed spatial differences consistent with those based on detection rates. Reaction times were shorter, on average, for targets in the lower versus the upper quadrants (p<0.01, one-way ANOVA followed by post hoc Tukey range test, n = 88 experiments across all birds). For all quadrants, reaction times decreased with increasing contrast (Figure 2D, robust regression, p<0.01 for slope, Bonferroni corrected for multiple comparisons). Contrast-response functions based on reaction times are shown for the individual birds in Figure S4.

The aspects of detection performance that differed across individual birds were their absolute and relative thresholds in each of the quadrants. We compared detection thresholds across birds with a two-way fixed-effects ANOVA (unbalanced design), with bird identity and visual field quadrant as categorical factors. The analysis revealed no main effect of bird identity (F(2, 297) = 3.0, p>0.05), indicating that no bird outperformed the others overall. However, the main effect of visual quadrant was strong (F(3, 297) = 49.4, p<0.001) as was the interaction effect (bird*quadrant, F(6, 297) = 10.8, p<0.001), indicating that different birds exhibited different thresholds in different quadrants (Figs. 2E,S3). Post-hoc tests, analyzing the data from individual birds separately, revealed that detection thresholds were lower in the lower quadrants versus the upper quadrants for two out of the three birds (p<0.01, Tukey range test). Moreover, they were lowest in the lower left quadrant versus the other 3 quadrants for all of the birds (p<0.01, Tukey range test; Figs. 2E,S3).

Inter-individual variability in reaction times was tested with a N-way fixed-effects ANOVA with contrast as a continuous factor, and bird and upper versus lower hemifield (left and right quadrant data pooled) as categorical factors. The analysis revealed no main effect of bird (F(2, 818) = 1.79, p>0.05), but an interaction effect of contrast*bird (F(2,818) = 6.6, p = 0.0014), indicating that while no bird reacted faster than the others overall, reaction times for each bird varied differently with target contrast (Figure S4). A main effect of hemifield (F(1,818) = 5.02, p = 0.025) and an interaction effect of hemifield*contrast (F(1,818) = 9.24, p = 0.0024) indicated that, overall, birds reacted with different speeds to targets in the upper versus lower hemifields, and that the reaction times varied systematically, but differently, with target contrast in the different hemifields (Figure S4). Other interaction effects were not significant (at the p = 0.05 level). Post-hoc analysis confirmed that marginal mean reaction times were faster for lower versus upper hemifield targets (Figure S4).

The data presented so far indicate that the chickens were more sensitive to targets in either of the lower quadrants than in either of the upper quadrants. We looked for evidence that detection performance in these pairs of quadrants was dynamically linked. We performed pair-wise correlations of detection thresholds (residuals; Materials and Methods) for each bird for each quadrant from each session. A correlation matrix of detection thresholds across visual quadrants revealed a striking correlation in performance between the two upper quadrants (R^2^ = 0.35, p<0.001, robust regression, Figure 2F, Figure S5A) and between the two lower quadrants (R^2^ = 0.47, p<0.001, Bonferroni corrected, Figure 2F, Figure S5B). Correlations between all other pairs of quadrants were not significant (p>0.05, Figure 2F, Figure S5C–E). This result demonstrates that detection performance was linked, session-by-session, across the upper quadrants and across the lower quadrants, respectively, but not across the hemifields.

Taken together, the data indicate that chickens exhibited a distinct advantage for detecting small targets in the lower visual field, an asymmetry that could reflect innate differences in visual sensitivity or response bias (see Discussion). In addition, target detection performance, which varied across test sessions, was dynamically linked within the upper and lower visual fields, respectively, and varied independently in the upper versus lower visual fields.

### Effect of Target Probability on Detection Performance

Humans are more sensitive to, and respond faster to, stimuli that appear with high probability at a particular location [8], [9]. We tested, in chickens, the effect of altering target probability on detection rates and reaction times in each of the four quadrants. In each block of 300–400 trials, targets appeared with high-probability (“HiP”; 80% of trials) in one of the four quadrants and with low probability (“LoP”; 20% of trials) in the diametrically opposed quadrant (Figure 3A, Materials and Methods). The tested pair of quadrants, corresponding to the HiP and LoP locations, was counterbalanced among the four quadrants across sessions.

Increased target probability improved detection performance dramatically for targets in the upper visual field quadrants. Figure 3B shows contrast-response functions from a representative bird for high and low probability targets in the upper hemifield. Contrast-response functions for HiP targets were systematically shifted towards lower target contrasts. Detection rates were consistently higher at all target contrasts for the HiP (thick line) compared to LoP (thin line) condition, and intermediate between the two for the EqP (dashed-line) condition.

Similar trends were observed across the population of birds. For the upper visual field quadrants, target detection rates were consistently higher in the HiP condition than in the EqP condition (Figure 3C): contrast-response functions and thresholds were shifted towards lower stimulus contrasts (p<0.05, Tukey range test, marginal mean contrast thresholds for HiP versus EqP, n = 80 experiments pooled across birds). Similarly, mean reaction times in the upper quadrants were lowest for the HiP condition (Figure 3D, p<0.05, Tukey range test, marginal mean reaction times for HiP versus EqP, pooled across birds). When contrast-response functions were measured in the LoP condition, the opposite effect was observed for targets in the upper visual field: detection performance was poorer than in either the HiP or EqP conditions (Figure 3C–D, p<0.01, Tukey range test, pooled across birds), and reaction times were longer. Analyzing the data for each bird separately revealed similar effects of spatial probability for targets in the upper hemifield (Figure S6).

Performance in the lower field quadrants was not significantly different between the HiP, EqP and LoP conditions in the population data both in detection rates and reaction times (Figure S7A, p>0.05, n = 80 experiments pooled across birds). No significant differences were found when the data were analyzed separately for individual birds (Figure S7B–C).

We observed an interesting corollary of the asymmetrical effect of target probability on target detection in the upper versus lower hemifields: when stimulus probability was high, detection performance was more uniform across the visual field. This effect is most apparent when data from the different quadrants are plotted on the same graph for each of the HiP and LoP conditions (Figure 3E). Quadrant-specific contrast-response functions measured in the HiP condition (Figure 3E, left; Figure S8A) were more clustered, compared with those measured in the LoP condition (Figure 3E, right; Figure S8B).

Thus, chickens exhibit enhanced detection performance at locations with high target probability, and the dynamic facilitatory effects of high spatial probability selectively improved target detection in the upper hemifield.

### Effect of Immediate History on Detection Performance

In the previous analysis, the modulatory effects of target spatial probability were measured over an entire block of trials. Next, we asked whether the bird’s recent performance history (successful detections) would predict modulations in detection performance on a finer, trial-by-trial timescale. To answer this question, we analyzed detection performance for targets in the upper hemifield, since the largest modulation of performance occurred selectively for upper hemifield targets (previous section).

**Figure 4:**
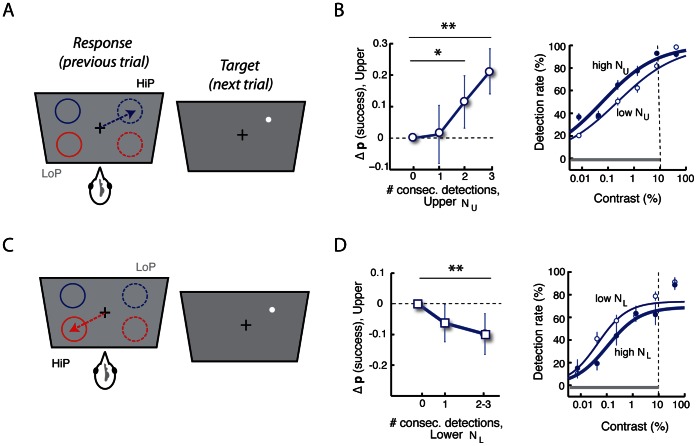
Target detection performance depends on recent history of detections. **A.** Task configuration for analyzing target detection performance in an upper quadrant as a function of the number of consecutive detections in the same quadrant. **B.** *(Left)* Change in the probability of detecting targets in the upper quadrants following 0,1, 2 or 3 consecutive successful detections (N_U_) in the same quadrant. After matching target contrasts (grey bar in right panel), the probability of a successful detection in the HiP quadrant increased significantly with more consecutive detections in the same quadrant (** p<0.01; * p<0.05, bootstrap test). Error bar: jackknife standard error. *(Right)* Contrast-response functions (detection rates) for targets following 0 (thin line) or 1–3 consecutive detections (thick line) in the same quadrant. **C.** Task configuration for analyzing target detection performance in an upper quadrant as a function of the number of consecutive detections in the opposite quadrant. **D.** *(Left)* Change in the probability of detecting targets in the upper quadrants following 0, 1 or 2–3 consecutive successful detections (N_L_) in the opposite quadrant. *(Right)* Contrast-response functions (detection rates) for upper hemifield targets following 0 (thin line) or 1–3 consecutive detections (thick line) at the opposite (lower hemifield) quadrant. Other conventions are the same as in (B).

First, we tested whether detection performance at an upper hemifield location would improve with the number of immediately preceding targets that were successfully detected at the same location (N_U_) (Figure 4A). Because the largest range of consecutive target presentations at a given location occurred during the HiP condition (2–6 consecutive targets), we measured detection performance in sessions where the HiP condition occurred in the upper hemifield. We analyzed the effect of N_U_ on detection performance while controlling for the effect of target contrast with two procedures: (a) a partial correlation analysis with target contrast as the confounding factor, and (b) computing average detection rates for trials after matching each N_U_ for the distribution of target contrasts, following a mean-matching procedure (Materials and Methods).

Partial correlations between the detection rate and N_U_, with target contrast as the confounding factor (Materials and Methods), revealed that detection rates improved (correlated positively) with increasing N_U_ in both of the upper quadrants (slope = 6.67%/trial, r_p_ = 0.28, p<0.001, pooled data). Similarly, reaction times decreased (correlated negatively) with increasing N_U_ (slope = −18.3 ms/trial, r_p_ = −0.13, p = 0.030, pooled data). Thus, a recent history of successful detections at a location improved the bird’s detection performance for subsequent targets at the same location.

We confirmed this finding with the mean-matching procedure (described above). Detection rates for a target in the upper hemifield improved significantly after one or more consecutively detected targets at the same location (Figure 4B, left; p<0.01, bootstrap test). This effect resulted in a leftward shift in the contrast-response function with increasing N_U_ (Figure 4B, right, thin line N_U = _0 versus thick line, N_U_ = 1–3). The difference in performance was most apparent in the low to intermediate range of target contrasts (Figure 4B, right).

We also asked the converse question: does improved detection performance at an upper hemifield location decrease with the number of successful detections at the opposite (lower hemifield) location? Again, as the greatest number of consecutive presentations occurred during the HiP condition, we measured detection performance in the upper hemifield during the LoP condition as a function of the number of consecutive successes in the lower hemifield during the HiP condition (N_L_). Detection rates decreased in the upper visual field with increasing N_L_ (Figure 4D, left, p<0.01, bootstrap test; Materials and Methods). Accordingly, contrast-response functions shifted to the right (to higher contrasts) for targets in the upper visual field in trials after 1–3 consecutive target detections in the lower visual field (Figure 4D, blue thin line N_L_ = 0 versus thick line, N_L_ = 1–3).

In contrast, detection performance for targets in the lower hemifield was only weakly modulated by detection history in the lower hemifield (Figure S9A), and was largely independent of detection history in the upper hemifield (Figure S9B). This result might be explained by the superior detection of targets in the lower hemifield at baseline (see Discussion).

The data demonstrate that detection performance in the upper visual field is strongly modulated, over short timescales, by the recent history of detections in both the upper and lower hemifields. Thus, chickens possess a mechanism that dynamically facilitates the detection of targets at a location with a history of successful detections, and inhibits detection at the opposite location.

## Discussion

In this study, we introduce a multiple alternative detection task based on a Go/NoGo paradigm that chickens learn rapidly and perform reliably. Unlike simpler Yes/No or two alternative forced choice tasks, this paradigm enables the measurement of target detection performance at several locations within a single experimental session. This paradigm permitted us to measure asymmetries in target detection in the upper vs. lower hemifields, and the modulation of detection performance by varying target spatial probability. How do these differences in detection performance arise, and what processes could contribute to this dynamic variation?

### Detection Asymmetries between the Upper and Lower Visual Hemifields

Chickens detected small stimuli faster and at lower contrasts in the lower than in the upper visual field locations. This lower visual field advantage, like the one reported in humans [2], [6], can be accounted for by the hypothesis that space-dependant differences in performance reflect consistent differences (over evolutionary time, or over an animal’s lifetime) in the spatial statistics of a behaviorally relevant feature across the visual field [6]. For chickens, the detection of small stimuli is relevant to foraging for food, a behavior both essential to survival and that occupies much of their waking time, and food items differentially occur in the lower visual field.

The lower visual field advantage for detecting small stimuli could result from a combination of visual, motor or attentional biases. The physiological basis for this lower visual field advantage remains to be determined. The advantage may reflect, in part, differences in the quality or quantity of the relevant retinal or motor circuitry or in the amount of representation devoted to the lower versus upper visual fields in sensorimotor brain areas that process small stimuli. Such differences have been demonstrated in the mammalian retina and brain [13], [14], as well as in the avian visual system [15], [16]. Our results document a clear advantage for locations in the lower hemifield. Although there was some inter-individual variability in detection thresholds in the lower-right quadrant, in all birds the lower-left quadrant exhibited the lowest contrast threshold. This result parallels previous findings of a left-sided visuospatial bias reported in birds [17], and specifically, an advantage for the lower-left quadrant observed in humans [18], [19]. In addition, birds responded fastest to stimuli in the lower quadrants, although there was some inter-individual variability in the magnitude of the difference in reaction times between upper and lower quadrants. This finding parallels the observation of a bias in humans for visually-guided movements in the lower visual field [2]. Further experiments are needed to test whether these advantages generalize across other kinds of stimuli and across other locations in the lower hemifield.

Detection thresholds fluctuated coherently in the two upper quadrants and in the two lower quadrants over sessions across different days, but the fluctuations were independent across the upper and lower hemifields. These findings suggest that, at baseline, processing for the lower field is decoupled from that for the upper field and that processing for each hemifield can be adjusted independently. This capacity may be critical when a chicken is simultaneously engaged in the detection of food in the lower hemifield and the detection of predators in the upper hemifield.

### High Spatial Probability Moderates Detection Asymmetries

Increasing the probability of occurrence of targets at a given location, increased stimulus detection over various timescales, and decreased differences in detection performance in the lower versus upper hemifield. This was due to a selective improvement in the detection of stimuli in the upper hemifield with increasing target probability. The absence of this effect in the lower hemifield suggests that the mechanism that improves performance was already engaged in the lower hemifield, even when stimuli were occurring with low probability in the lower hemifield [20].

In addition, detection performance was modulated trial-by-trial by the recent history of target detections, an effect that is well documented in humans [21]. This mechanism improved performance at locations where stimuli had been detected in the immediately preceding trials, and impaired performance with successive detections at the diagonally opposite location. These data indicate a competitive process that operates across space on a short timescale. Thus, chickens are not only able to take advantage of the spatial distribution of sensory stimuli, they also are able to dynamically alter their performance at specific locations based on recent detection history.

### Mechanism Underlying Spatial Probability Effects

The task paradigm employed by this study is similar to those that have been used to measure attention in humans. The cue that we manipulated in this study was the probability that a target would occur at a particular location. For humans, this cue is thought to draw spatial attention to the high probability location, because detection improves and reaction times decrease for stimuli at that location [7], [9], [10]. For humans, as for chickens, this effect is particularly strong for low contrast stimuli, and it is spatially competitive, with stimulus detection deteriorating at other locations as detection improves at the high probability location. Could the modulations in detection performance reported in this study reflect the effects of spatial attention in chickens?

The improvements in detection performance with increased spatial probability observed in our task could correspond to enhanced visual sensitivity, or could represent a combination of motor or attention biases. Answering this question requires decoupling the effects of perceptual sensitivity from response bias in multiple alternative tasks. Rigorously addressing this challenge requires the development of new theoretical models (such as with signal detection theory [22]). Nevertheless, the shapes of the contrast-response functions provide preliminary empirical evidence to address this question. Contrast-response functions measured in the high spatial probability condition were not simply shifted toward lower contrasts relative to those measured in the low spatial probability condition (a bias effect), they also exhibited a steeper slope at threshold (Figure 3B–C), indicating that visual sensitivity was increased at the location of high spatial probability. This observation (increase in sensitivity or resolution), coupled with the observation of decreased reaction times to high probability locations (Figure 3D), parallel the behavioral effects of attentional cues in human tasks.

A variant of the behavioral paradigm that was employed in this study could be used to test this “attention” hypothesis. Stimulus probability, which acted as the spatial cue in this study, could be replaced with an explicit spatial cue that reliably predicts the location of a subsequent target stimulus. Such explicit spatial cueing is known to produce more robust effects in spatial attention tasks, at least in humans [8]. In order to employ such a paradigm with chickens, at least two criteria must be fulfilled: First, birds need to be able to learn to associate a spatial cue with the likelihood of a stimulus appearing at a specific location; and second, they must to be able to shift the locus of attention rapidly based on the location of the spatial cue. The findings from this study that chickens are able to utilize target spatial probability to improve performance at specific locations and rapidly update detection behavior based on recent history, suggest that chickens can, indeed, be trained to perform an explicit spatial cueing paradigm. The multiple alternative detection paradigm introduced by this study enables rigorous measurement of detection performance simultaneously at multiple locations in this non-mammalian species.

## Materials and Methods

### Ethics Statement

All procedures were in compliance with the guidelines of the National Institutes of Health for the care and use of laboratory animals and were approved by the Institute Animal Care and Use Committee of Stanford University (Protocol ID: 20287).

### Birds

Experiments were performed with three adult (>8 months) chickens. The birds were put on a food-restricted schedule until they reached 70% (∼1000 g) of their free feeding weight (∼1400 g). Birds were weighed before and after each training and testing session to ensure that they remained at 70–80% of their free-feeding weight. Water was always available in the home cage without restriction.

### Apparatus

Behavior was tested with a custom-built apparatus (Movie S1). The apparatus was housed in a sound chamber (3×3×2.5 m) with light, temperature and ventilation control. A large plexiglass box (.5×.5×1 m) at the center of the chamber contained a touch-sensitive computer screen (Acoustic Pulse Recognition Technology, Elo Touch Systems), a custom-built automatic feeder, infra-red video camera (NightVision, Sony®, USA), and two adjustable vertical posts in front of the screen. The chicken was placed into the box, and the floor of the box was adjusted so that the shoulder of the bird was even with the center of the computer screen. Stimulus presentation and data acquisition were automated by custom Matlab® (Natick, MA) scripts using the Psychophysics Toolbox extensions [23]. The experimenter sat at a console outside of the sound chamber to monitor the bird on a TV screen. During experiments, the chamber was dark and the apparatus was illuminated with infrared light.

### Training

We presented briefly-flashed dot stimuli, 3° in diameter at fixed spatial locations in the bird’s visual field. Here, and in the text, spatial locations and dimensions of the targets are defined relative to the mid-sagittal plane and the horizontal plane that contains the optical axes of the eye and the tip of the beak.

To begin a trial, a small (±5 mm) cross-hair appeared at the center of the computer screen. In order to peck on the cross-hair, the chicken had to position its body between the two vertical posts; this forced the body into a standard position relative to the screen. The chicken had to peck accurately on the cross-hair (within 10 mm) to initiate a trial; this forced the head to a standard position relative to the screen for 250–300 ms (Figure S1). Immediately after the peck on the cross-hair, the stimulus appeared on the screen for 50 ms. The stimulus disappeared before the chicken had a chance to move from the standard position (the earliest movement onsets occurred at latencies of well over 200 ms, Figure S1). Pecking is a stereotyped action pattern of binocular, frontal fixation during which the eyes assume a standard position in the head [24], [25]. Therefore, by monitoring head orientation (and thereby eye orientation) stimuli could be presented at consistent locations in the bird’s visual field. If the animal detected a target stimulus (Go trial), it pecked on the location where the target had appeared, causing the feeder to open for 2 s. If no target was detected (NoGo trial), it pecked twice on the cross-hair, causing the feeder to open for 2 s (Movie S1). The data from each trial consisted of a file containing the stimulus conditions and the timing and accuracy of pecks on the cross-hair and target.

Our objective was to develop a task that would measure detection performance at various locations in the bird’s visual field simultaneously. Hence, we developed a version of the multiple alternative detection task modeled on primate tasks. During training, NoGo responses to Go trials (misses) were not rewarded. However, Go responses to NoGo trials (false alarms), were severely penalized with a 30–60 s timeout. This was done to ensure that birds would never guess on NoGo trials in order to minimize the rate of false alarms. Birds were trained until they performed with over 90% accuracy on the Go trials and ∼100% accuracy on the NoGo trials for full contrast dots presented in all four visual quadrants.

We used only positive contrast dots (bright dots on a dark background) in all of these experiments. This permitted us to compare our results with previous findings in human and primate experiments [9], [10]. Moreover, we observed that chickens naturally preferred to peck on bright objects on a dark screen, rather than vice versa. In addition, a dark background provided low ambient light levels that permitted birds to stay on task by limiting the illumination of potentially distracting objects in the behavior chamber. Visual contrasts, expressed as luminance (in cd/m^2^) above background relative to the full range of luminances presented, were calibrated with a spectrometer (OceanOptics Inc.).

### Protocol for Mapping the Visual Field

The target stimulus was chosen to be a full contrast (bright) stationary dot, 3° in diameter. The target dot appeared immediately following the peck on the cross-hair, for 50 ms, at one of several azimuths and elevations (eccentricities of 40° to 70° in steps of 3–5°, and radial steps of 10–15°). The sequence of locations at which dots were presented occurred was pseudorandomized. While no NoGo trials were included in this testing, the rigorous training (outlined previously) ensured that birds reliably reported their inability to detect the appearance of a dot with a NoGo response.

The visibility of the stimulus across the visual field was assessed by the proportion of targets correctly detected and detection rates, averaged across birds. For plotting detection performance as a continuous function of visual space, detection rates were Delaunay interpolated using the TriScatteredInterp function in Matlab. Detection rates and reaction times were pooled from each hemifield (left versus right, or upper versus lower) and the medians of these distributions were compared across hemifields with a non-parametric statistical test (Wilcoxon signed rank test).

### Protocol for Measuring Contrast–response Functions

The target was identical to the previous protocol (positive contrast, stationary, 3° diameter dot, flashed for 50 ms). The target appeared at one of four possible locations: 60° in azimuth and 60° in elevation from the zeroing cross-hair in each of the four visual field quadrants. Targets appeared with equal probability at any of the four locations, and the sequence of target locations was pseudorandomized. We call this the “EqP” (equal probability) condition.

Detection rates (hits) as a function of target contrast were measured for each of the cued conditions and for each quadrant. Detection was measured with a staircase procedure, based on responses to blocks of trials. Each block consisted of 4 trials: 2 Go trials (of the same cued condition) and 2 NoGo trials. The NoGo trials tested whether the bird was performing the Go/NoGo task accurately. If the bird responded correctly to either of the Go trials with a Go response, then the contrast of the target was halved for that condition in that quadrant in the next block. If the bird gave a NoGo response to both Go trials, then the contrast of the target was doubled for that quadrant in the next block (Figure S2). Go and NoGo trials were randomly interleaved within a session. Detection performance was monitored on-line for each quadrant. Performance typically asymptoted within 10–15 blocks. After 10–15 blocks, the contrast of the target returned to full contrast, and the staircase was restarted. This procedure assured adequate sampling of high contrast targets, and kept the birds from becoming frustrated with low contrast targets. For ∼50% of the sessions, staircase functions were obtained with contrast decrements that were 3× larger (when the bird responded correctly) than contrast increments (when the bird gave an incorrect response). This permitted a rapid approach to asymptotic performance, and the sampling of contrast space at a finer resolution. Contrast-response functions were based on percent correct responses. We also measured response latencies as a function of target contrast.

To ensure that birds were performing consistently on NoGo trials, only experiments in which performance on the NoGo trials was better than 95% correct (fewer than 5% false alarms) were included in the analysis. For all tasks, unless otherwise indicated, data from each quadrant were treated separately.

Contrast-response curves were created using the following procedure: Detection rates based on % correct (hit rates) were first obtained for each testing session separately by binning contrasts in fixed logarithmic increments. These data were then combined across sessions and birds to produce the mean and error bars for the detection rates. Error bars were computed as the standard error of the mean detection rate for targets within a specific contrast bin across experimental sessions. Detection rates for targets of various contrasts were fit with a sigmoidal function of contrast.
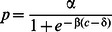
where p is the proportion of targets correctly detected as a function of target contrast, c; α is the asymptotic performance at full contrast; δ is the contrast at 50% performance, and β is the slope of the curve. Curve fits were obtained with the *lsqcurvefit* function in Matlab. Test sessions in which the data were fit poorly by these curves (R^2^<0.5) were excluded from the analyses. Mean R^2^ values were typically ∼0.85–0.90.

### Protocol for Measuring the Effects of Target Probability

The apparatus and methodologies for measuring the effects of target spatial probability were the same as those for measuring spatial asymmetries in contrast sensitivity (see above). The main difference was that the probability that the target appeared at a particular location was increased to 80% (high probability, HiP) for one of the four visual quadrants and was reduced to 20% (low probability, LoP) for the opposite quadrant. Test sessions were run in 2 periods of ∼300–400 trials separated by a 1 hr rest period. During the first period, the high probability location was in one of the four quadrants and the low probability location was in the diagonally opposed quadrant. During the second period, the high and low probability quadrants were switched. Again, the staircase procedure was used to assess the effects of target contrast on detection performance.

We hypothesized that the effects of target spatial probability would be diminished by interrupting the sequential presentation of stimuli at a particular location with NoGo trials, a large percentage (50%) of which were interleaved with the stimulus presentation trials. Therefore, in a subset test blocks only a few NoGo trials were included: NoGo trials were only presented when the birds appeared to detect even the smallest contrast that the screen could display, in order to ensure that birds were not guessing. Repeating the analysis with these blocks alone revealed qualitatively similar, but stronger effects of target probability, as expected.

### Statistical Analysis of Contrast Thresholds and Reaction Times

For the EqP condition, Contrast thresholds (δ, contrast at 50% performance) for data pooled across birds were analyzed with a two-way, fixed-effects ANOVA (unbalanced design), with bird identity and visual field quadrant as categorical factors. To assess inter-individual variability, post-hoc testing was performed with the Tukey range test followed correction for multiple comparisons (Tukey HSD) (*multcompare* function in Matlab). To assess differences in reaction time with location across the population of birds, mean reaction times (across all target contrasts) for each session were compared with a two-way ANOVA. To assess inter-bird variability, reaction times were tested with an N-way, fixed-effects ANOVA with contrast as a continuous factor, and bird and upper versus lower hemifield (left and right quadrant data pooled) as categorical factors. Post-hoc analyses were performed with the Tukey range test. For comparing detection performance across the HiP, EqP and LoP conditions, contrast thresholds and mean reaction times were subjected to an N-way ANOVA with bird identity, condition (HiP, EqP, LoP), and visual hemifield (upper versus lower) as categorical factors followed by a Tukey range test for multiple comparison correction.

### Correlation Analysis of Contrast Thresholds between the Quadrants

We performed pair-wise correlations of detection thresholds for each bird for each quadrant from each session. In order to pool the data across birds, these correlations were performed after subtracting the mean threshold for each quadrant for each bird (due to the strong interaction effect of bird*quadrant). R^2^ and p-values of detection thresholds (residuals) between each pair of quadrants were computed with robust regression (*robustfit* function in Matlab), and Bonferroni corrected for multiple comparisons.

### Statistical Analysis of Recent History Effects on Detection Performance

This analysis was performed to test whether the number of targets consecutively detected (N_X_) at a location X affected the detection of a target subsequently presented at (a) the same or (b) opposite location. Data in the upper and lower hemifield quadrants were pooled across birds. As the greatest number of consecutive presentations occurred during the HiP condition, we measured detection performance in the upper hemifield as a function of the number of consecutive successes in the HiP condition at either the upper (N_U_), or the lower hemifield (N_L_) locations.

To control for any systematic effects of target contrast, we employed a partial-correlation measure that quantifies the degree of association between two random variables, while removing the effects of a set of control variables. Partial correlations were computed between the detection rates or reaction times and N_X_, with target contrast as the confounding factor, with the *partialcorr* function in Matlab, and the fits to the residuals (slopes, p-values) were generated with the *addedvarplot* function.

To control for the effects of target contrast more stringently, we adopted a mean matching procedure for contrasts. Contrast space was binned into narrow logarithmic bins (bin width was roughly 2% of the range of the tested contrasts), and detection successes and failures within each of these contrast bins were pooled. For each N_X_, mean detection rates were computed by averaging performance across the lowest common number of trials for each contrast bin, and the mean performance was averaged across contrasts. If no trials existed within a contrast bin for any value of N_X_, performance in that range of contrasts was excluded form averaging. The distribution of contrasts matched for each analysis is shown in the respective figure with a grey horizontal bar (Figs. 4B, D right). Jackknife variance estimates were computed for performance at each N_X_ across sessions by repeatedly re-computing performance after leaving out one session in turn. To test for significant differences in performance between each pair of N_X_, we used a bootstrap procedure [26]. Null distributions were computed with the bootstrap method by randomly interchanging N_X_ labels and re-computing the difference in detection rates between pairs of N_X_ values. This procedure was repeated 1000 times. One-tailed p-values correspond to the fraction of values in the bootstrap distribution that exceeded the experimentally observed detection rate differences.

## Supporting Information

Figure S1 **Head trajectory of a chicken following a peck. A.** Representative traces from >100 trials (bird 1) showing the displacement of the head in the X (top), Y (middle) and Z (bottom) dimensions relative to its initial position following a peck. t = 0 ms corresponds to time of contact of the beak with a touch-sensitive screen (X: left vs. right axis of motion, parallel to the screen; Y: front vs. back axis of motion, perpendicular to the screen; Z: up vs. down axis of motion, perpendicular to the floor). **B.** Root-mean-square (rms) displacement of the head showing its distance from its initial position following the peck. *Thin lines*: standard error of the mean. These data represent the stereotypical trajectory of head movements following the peck, and demonstrate that the head remains fairly stationary for ∼250–300 ms after the peck. Head position was tracked with infrared reflective markers mounted on the head (OptiTrack, Natural Point).
(PDF)

Figure S2 **Staircase plots from a representative EqP session.** Staircase plots showing the progression of target contrasts (y-axis) over blocks (x-axis) in each visual quadrant, for a representative session (bird 3) in the EqP condition (inset). Each block comprised 2 Go and 2 NoGo trials. Success in at least one Go trial resulted in a decrement in target contrast in the next block, whereas failure in both Go trials resulted in a contrast increment. Failure in any NoGo trial (false-positive) terminated the block. Target contrasts were stair-cased independently in the four quadrants. Blue: upper hemifield quadrant. Red: lower hemifield quadrant. Left and right panels show left and right quadrant data, respectively. Dotted grey line: lowest contrast targets tested. Dotted black line: zero-contrast.
(PDF)

Figure S3 **Contrast-response functions based on detection rates in the EqP condition. A.** Contrast-response functions based on detection rates in the EqP condition for each individual bird. Columns: individual bird data. Other conventions are the same as in Figure 2C (main text). **B.** Mean contrast thresholds for the different visual quadrants in the EqP condition for each individual bird. Other conventions are the same as in Figure 2E (main text).
(PDF)

Figure S4 **Contrast-response functions based on reaction times in the EqP condition.** *(All panels)* Blue data: upper hemifield. Red data: lower hemifield. **A.** *(Left)* Reaction times in the upper and lower quadrants as a function of contrast in the EqP condition (data pooled across the left and right hemifield quadrants and across birds). *(Right)* Mean reaction times, averaged across targets of all contrasts. Error bars denote standard error of the mean across sessions. Mean reaction times were significantly lower in the upper relative to the lower hemifield (p<0.01, ANOVA, n = 88 experiments in 3 birds). **B.** Reaction times as a function of contrast for each individual bird in the EqP condition. Reaction times tended to decrease linearly with target contrast. Columns: individual bird data. Other conventions are the same as in (A, left). **C.** Mean (marginal) reaction times for each individual bird in the EqP condition. Mean reaction times were significantly lower in the upper relative to the lower hemifield for all birds (* p<0.05; ** p<0.01, ANOVA). Columns: individual bird data. Other conventions are the same as in (A, right).
(PDF)

Figure S5 **Pair-wise correlations of contrast thresholds between the visual quadrants. A.** Distribution of contrast thresholds (residuals) in the upper hemifield quadrants measured in each test session in the EqP condition. Contrast thresholds were positively correlated between the upper hemifield quadrants across test sessions (see main text). Data from each bird are indicated with a different symbol. **B.** Same as in (A) but for the lower-hemifield quadrants (left vs. right). **C.** Same as in (A) but for the right-hemifield quadrants (upper vs. lower). **D.** Same as in (C) but for the left-hemifield quadrants (upper vs. lower). **E.** p-values corresponding to the pair-wise correlation matrix (Figure 2F, main text). Warmer shades correspond to lower p-values (higher significance levels).
(PDF)

Figure S6 **Detection rates and reaction times in the HiP and LoP conditions for the upper hemifield locations. A.** Contrast-response functions based on detection rates for the HiP and LoP conditions in the upper hemifield for individual birds. Filled circles and thick line: detection rates and sigmoidal fit for the HiP condition. Open circles and thin line: detection rates and sigmoidal fit for the LoP condition. Columns: individual bird data. **B.** Mean (marginal) reaction times for individual birds in the HiP and LoP conditions in the upper hemifield. Filled bars: HiP condition. Open bars: LoP condition. Columns: individual bird data. Other conventions are the same as in Figure S4.
(PDF)

Figure S7 **Detection rates and reaction times in the HiP and LoP conditions for the lower hemifield locations. a.** *(Left)* Contrast-response functions (population summary data) based on detection rates for lower hemifield targets for the HiP and LoP conditions. Other conventions are the same as in Figure 3C. *(Right)* Contrast-response functions (population summary data) based on reaction times for lower hemifield targets for the HiP and LoP conditions. Other conventions are the same as in Figure 3D. **b.** Same as figure S6A, but detection rates for lower hemifield targets for individual birds. **c.** Same as figure S6B, but reaction times for lower hemifield targets for individual birds.
(PDF)

Figure S8 **Contrast-response functions in the HiP and LoP conditions. A.** Contrast-response functions based on detection rates for the HiP condition in each visual quadrant for individual birds. Columns: individual bird data. Other conventions are the same as in Figure 3E (main text). **B.** Same as in (A) but for the LoP condition. Other conventions are the same as in (A).
(PDF)

Figure S9 **Variation of detection performance in the lower hemifield locations based on recent detection history. A.** Task configuration for analyzing target detection performance in a lower quadrant as a function of the number of consecutive detections in the same quadrant. **B.** *(Left)* Change in the probability of detecting targets in the upper quadrants following 0,1, 2 or 3 consecutive successful detections (N_L_) in the same quadrant. Other conventions are the same as in Figure 4B. **C.** Task configuration for analyzing target detection performance in a lower quadrant as a function of the number of consecutive detections in the opposite quadrant. **D.** *(Left)* Change in the probability of detecting targets in the upper quadrants following 0, 1 or 2–3 consecutive successful detections (N_U_) in the opposite quadrant. Other conventions are the same as in Figure 4D.
(PDF)

Movie S1 **A chicken performing a multiple-alternative task.** A chicken performs a multiple alternative task involving the detection of a briefly-flashed target. The bird pecks on a zeroing cross to initiate the trial and, immediately afterward, a target (small dot) is briefly flashed (50 ms) on the screen. In the first trial, the target appears in the upper right quadrant, and in the second trial, in the lower left quadrant of the bird’s visual field. In these trials, the bird is rewarded for pecking at the location of the target (Go response). In the third trial, no dot is presented, and the bird is rewarded for pecking once more on the zeroing cross (NoGo response). The screen turns bright to signal the end of each trial, and to provide sufficient illumination for the bird to feed.
(MP4)
