# Supplementary figures and images for: Spatial Probability Dynamically Modulates Visual Target Detection in Chickens
Source: PLoS One. 2013 May 29;8(5):e64136. doi: 10.1371/journal.pone.0064136 (PMC3667102; doi:10.1371/journal.pone.0064136)

Figure S1

A

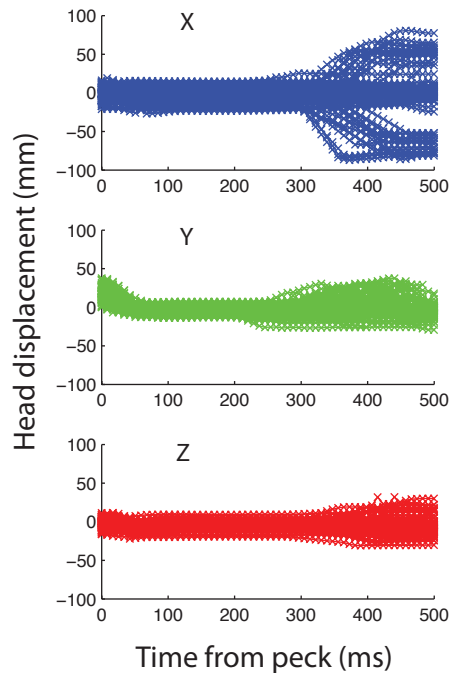

B

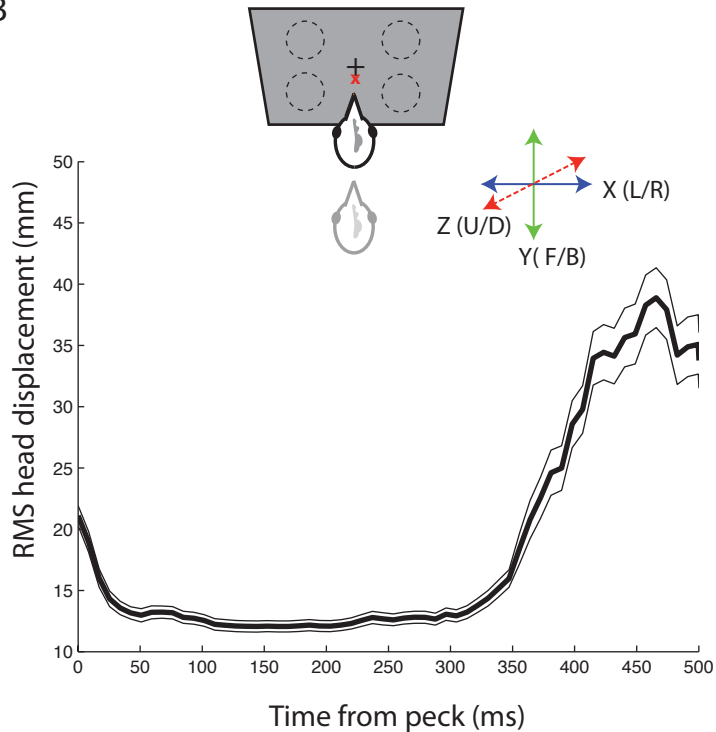

Supplement: Figure S1 — Head trajectory of a chicken following a peck. A. Representative traces from >100 trials (bird 1) showing the displacement of the head in the X (top), Y (middle) and Z (bottom) dimensions relative to its initial position following a peck. t = 0 ms corresponds to time of contact of the beak with a touch-sensitive screen (X: left vs. right axis of motion, parallel to the screen; Y: front vs. back axis of motion, perpendicular to the screen; Z: up vs. down axis of motion, perpendicular to the floor). B. Root-mean-square (rms) displacement of the head showing its distance from its initial position following the peck. Thin lines: standard error of the mean. These data represent the stereotypical trajectory of head movements following the peck, and demonstrate that the head remains fairly stationary for ∼250–300 ms after the peck. Head position was tracked with infrared reflective markers mounted on the head (OptiTrack, Natural Point). (PDF) [file pone.0064136.s001.pdf]

Figure S2

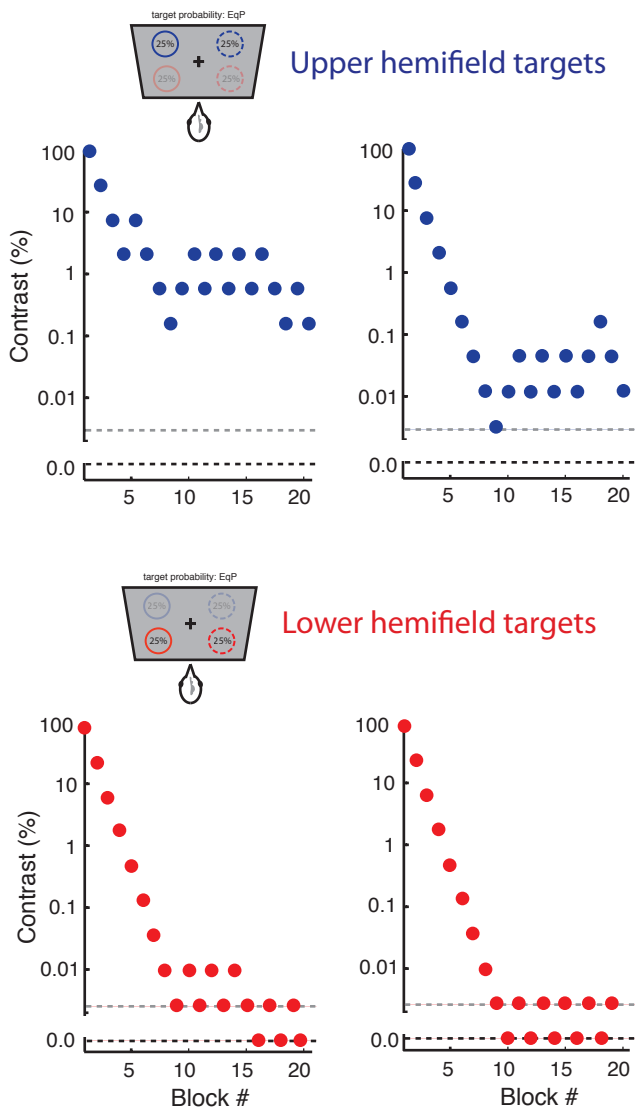

Supplement: Figure S2 — Staircase plots from a representative EqP session. Staircase plots showing the progression of target contrasts (y-axis) over blocks (x-axis) in each visual quadrant, for a representative session (bird 3) in the EqP condition (inset). Each block comprised 2 Go and 2 NoGo trials. Success in at least one Go trial resulted in a decrement in target contrast in the next block, whereas failure in both Go trials resulted in a contrast increment. Failure in any NoGo trial (false-positive) terminated the block. Target contrasts were stair-cased independently in the four quadrants. Blue: upper hemifield quadrant. Red: lower hemifield quadrant. Left and right panels show left and right quadrant data, respectively. Dotted grey line: lowest contrast targets tested. Dotted black line: zero-contrast. (PDF) [file pone.0064136.s002.pdf]

Figure S3

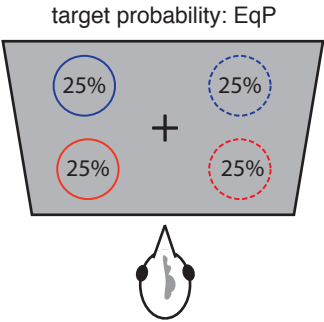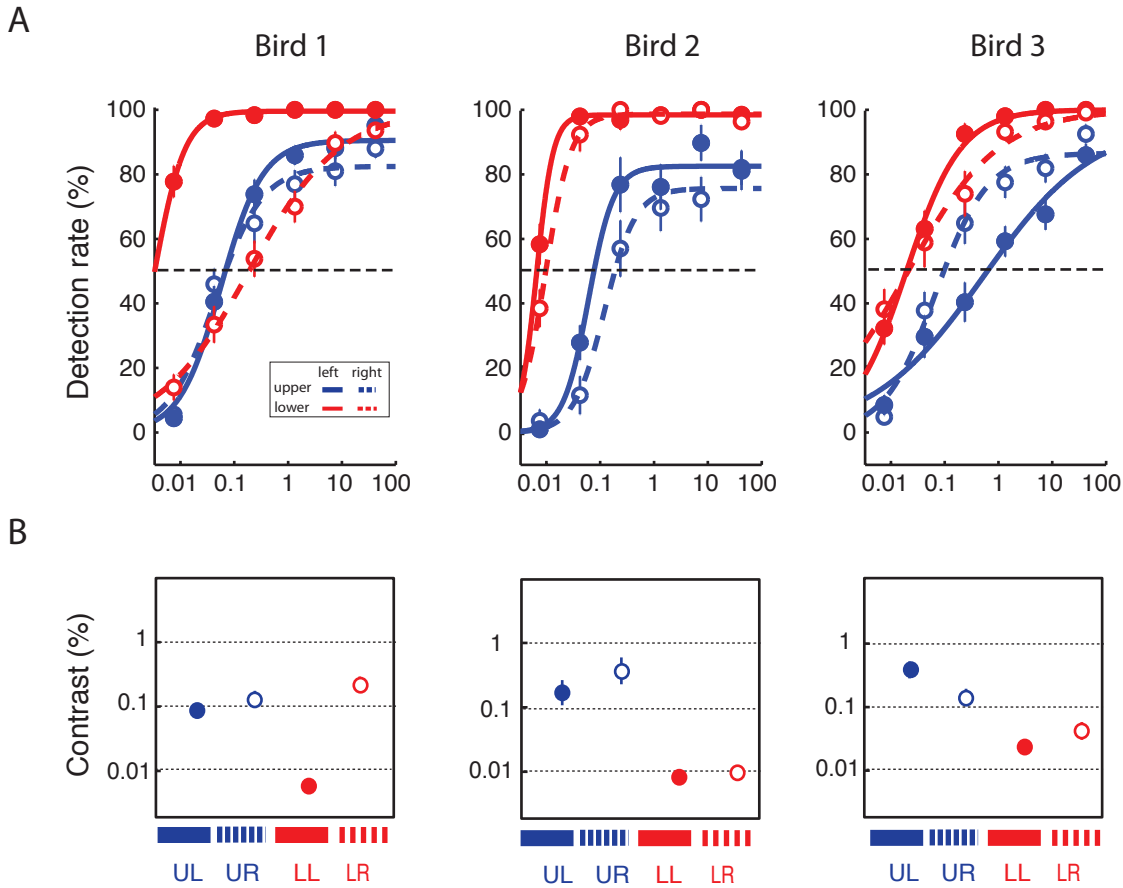

Supplement: Figure S3 — Contrast-response functions based on detection rates in the EqP condition. A. Contrast-response functions based on detection rates in the EqP condition for each individual bird. Columns: individual bird data. Other conventions are the same as in Figure 2C (main text). B. Mean contrast thresholds for the different visual quadrants in the EqP condition for each individual bird. Other conventions are the same as in Figure 2E (main text). (PDF) [file pone.0064136.s003.pdf]

Figure S4

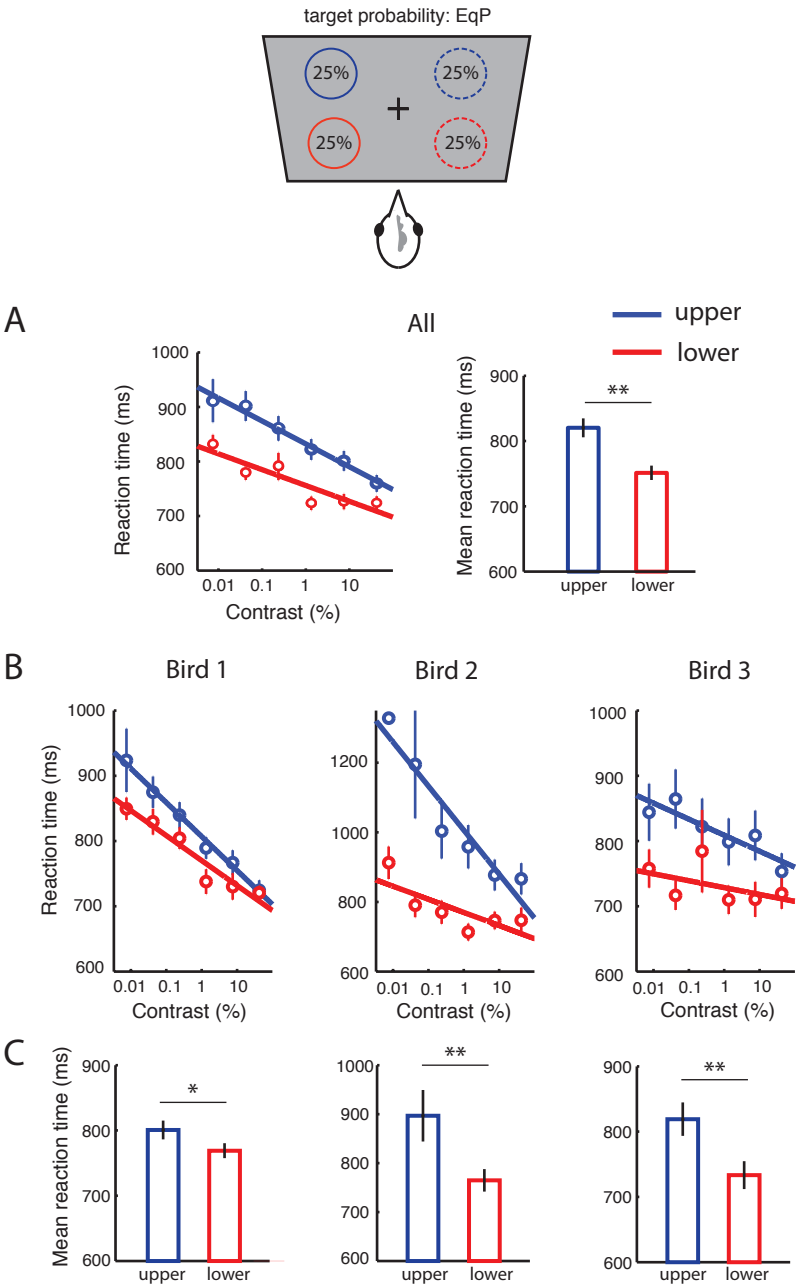

Supplement: Figure S4 — Contrast-response functions based on reaction times in the EqP condition. (All panels) Blue data: upper hemifield. Red data: lower hemifield. A. (Left) Reaction times in the upper and lower quadrants as a function of contrast in the EqP condition (data pooled across the left and right hemifield quadrants and across birds). (Right) Mean reaction times, averaged across targets of all contrasts. Error bars denote standard error of the mean across sessions. Mean reaction times were significantly lower in the upper relative to the lower hemifield (p<0.01, ANOVA, n = 88 experiments in 3 birds). B. Reaction times as a function of contrast for each individual bird in the EqP condition. Reaction times tended to decrease linearly with target contrast. Columns: individual bird data. Other conventions are the same as in (A, left). C. Mean (marginal) reaction times for each individual bird in the EqP condition. Mean reaction times were significantly lower in the upper relative to the lower hemifield for all birds (* p<0.05; ** p<0.01, ANOVA). Columns: individual bird data. Other conventions are the same as in (A, right). (PDF) [file pone.0064136.s004.pdf]

Figure S5

A

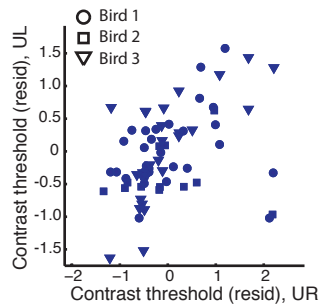

B

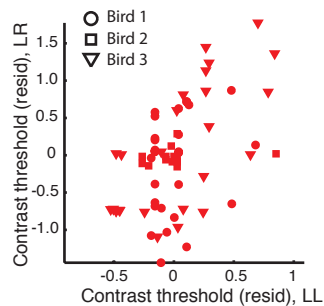

C

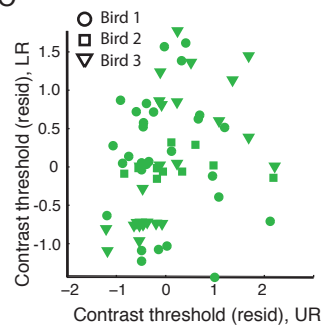

D

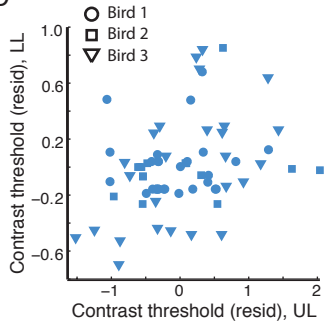

E

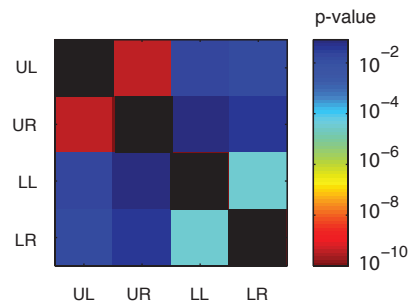

Supplement: Figure S5 — Pair-wise correlations of contrast thresholds between the visual quadrants. A. Distribution of contrast thresholds (residuals) in the upper hemifield quadrants measured in each test session in the EqP condition. Contrast thresholds were positively correlated between the upper hemifield quadrants across test sessions (see main text). Data from each bird are indicated with a different symbol. B. Same as in (A) but for the lower-hemifield quadrants (left vs. right). C. Same as in (A) but for the right-hemifield quadrants (upper vs. lower). D. Same as in (C) but for the left-hemifield quadrants (upper vs. lower). E. p-values corresponding to the pair-wise correlation matrix (Figure 2F, main text). Warmer shades correspond to lower p-values (higher significance levels). (PDF) [file pone.0064136.s005.pdf]

Figure S6

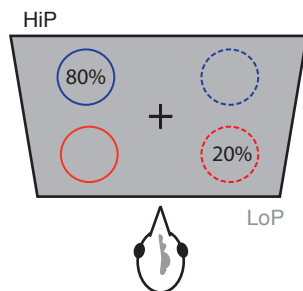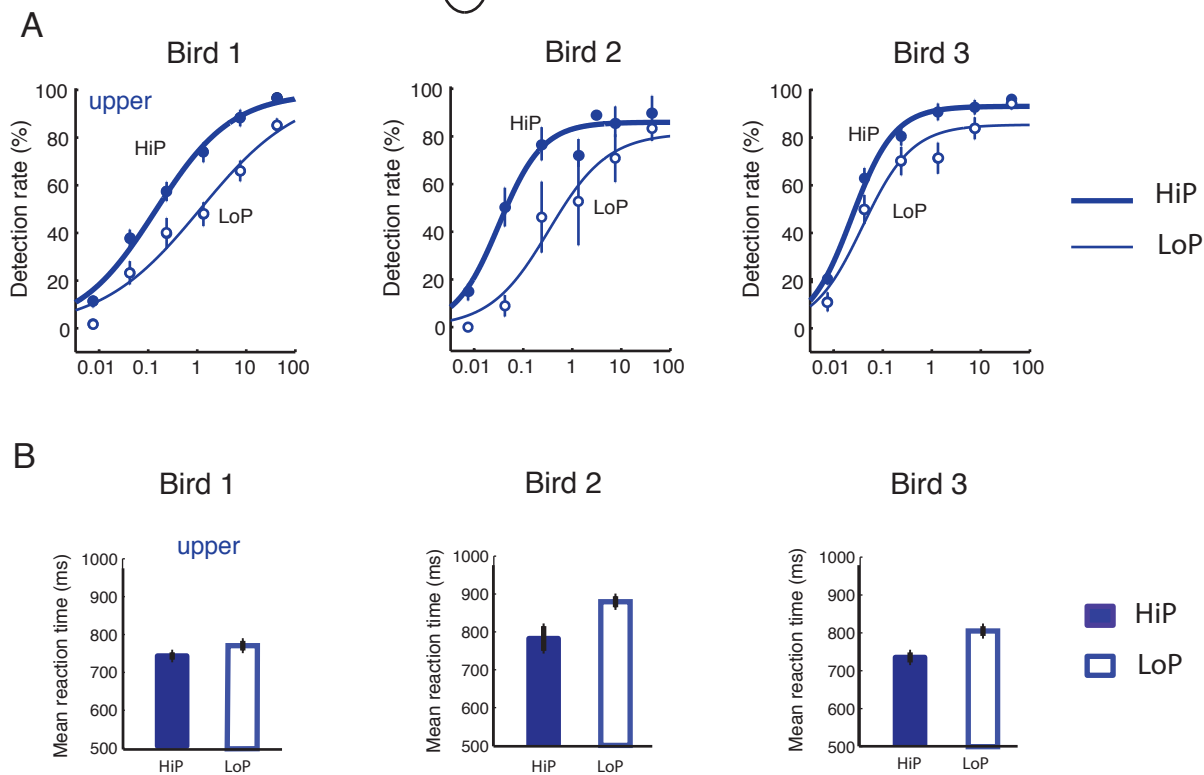

Supplement: Figure S6 — Detection rates and reaction times in the HiP and LoP conditions for the upper hemifield locations. A. Contrast-response functions based on detection rates for the HiP and LoP conditions in the upper hemifield for individual birds. Filled circles and thick line: detection rates and sigmoidal fit for the HiP condition. Open circles and thin line: detection rates and sigmoidal fit for the LoP condition. Columns: individual bird data. B. Mean (marginal) reaction times for individual birds in the HiP and LoP conditions in the upper hemifield. Filled bars: HiP condition. Open bars: LoP condition. Columns: individual bird data. Other conventions are the same as in Figure S4. (PDF) [file pone.0064136.s006.pdf]

Figure S8

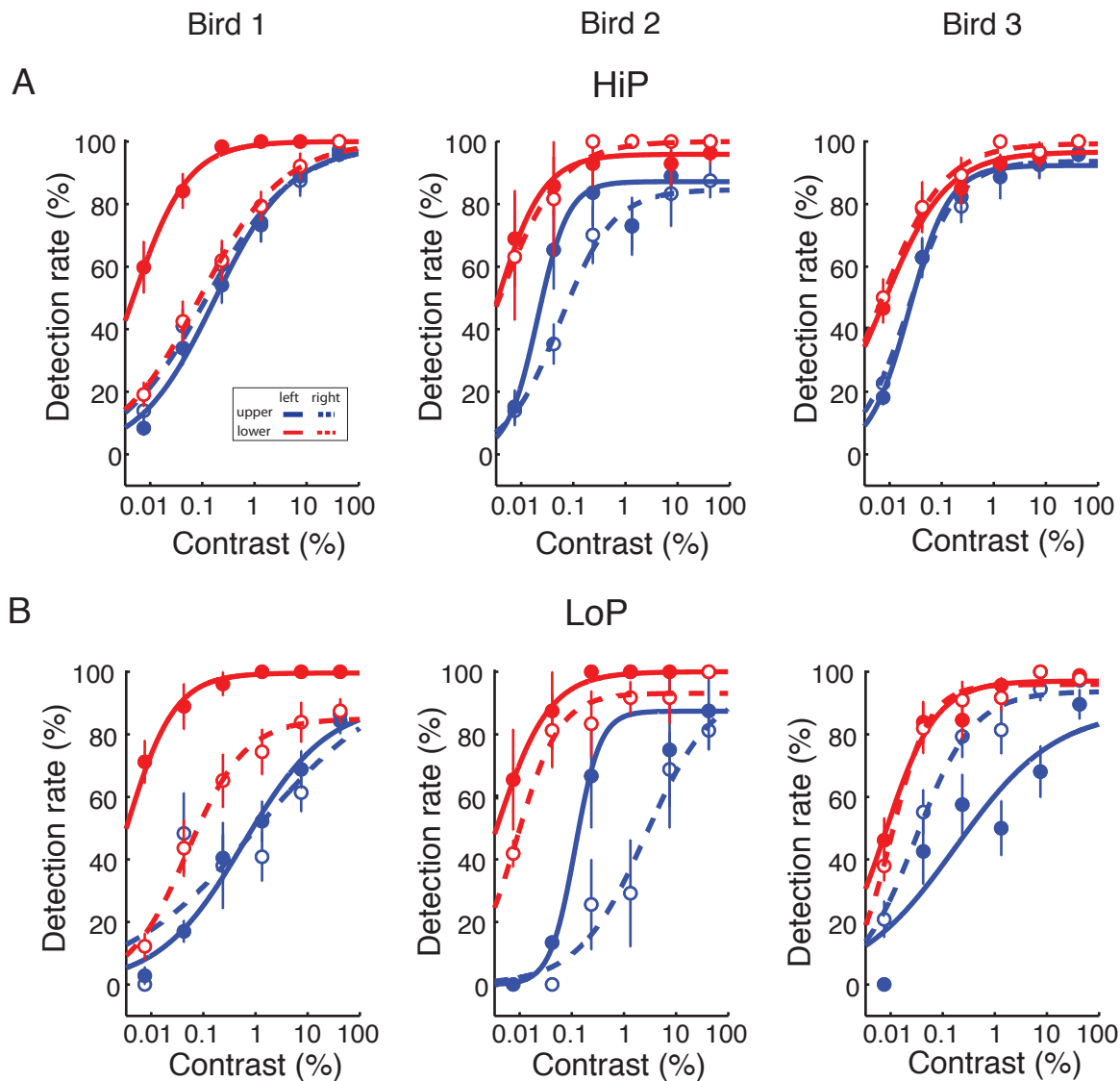

Supplement: Figure S8 — Contrast-response functions in the HiP and LoP conditions. A. Contrast-response functions based on detection rates for the HiP condition in each visual quadrant for individual birds. Columns: individual bird data. Other conventions are the same as in Figure 3E (main text). B. Same as in (A) but for the LoP condition. Other conventions are the same as in (A). (PDF) [file pone.0064136.s008.pdf]

Figure S9

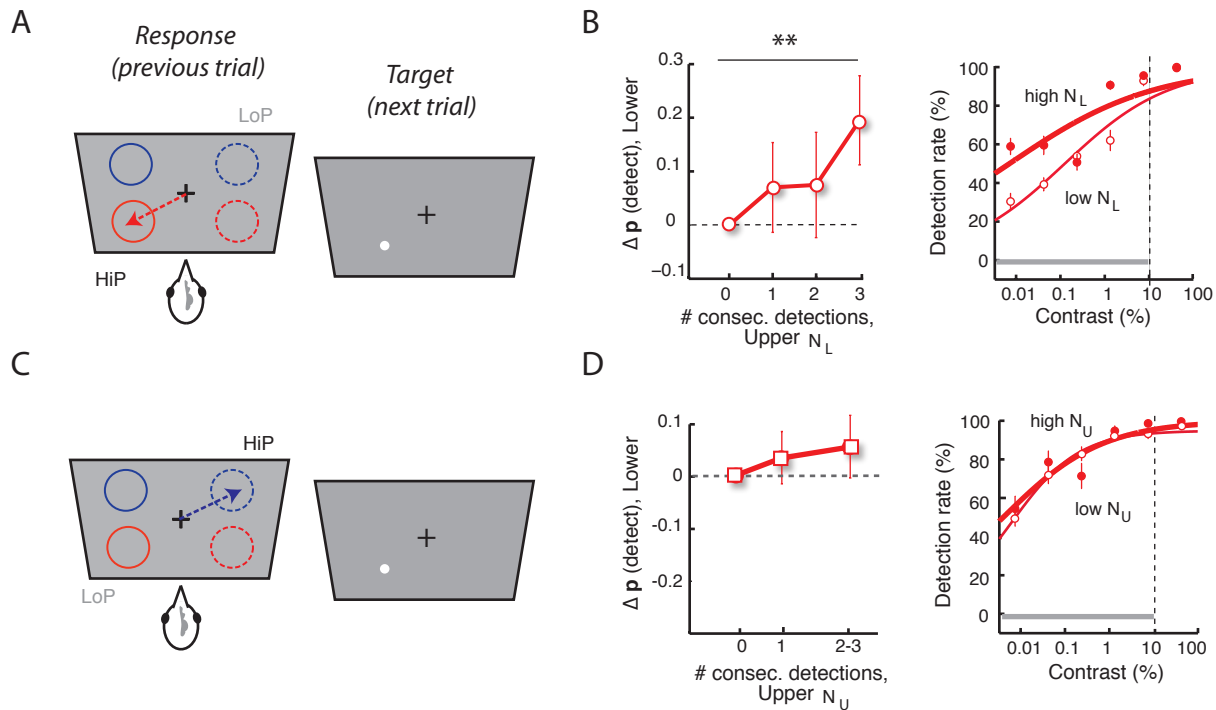

Supplement: Figure S9 — Variation of detection performance in the lower hemifield locations based on recent detection history. A. Task configuration for analyzing target detection performance in a lower quadrant as a function of the number of consecutive detections in the same quadrant. B. (Left) Change in the probability of detecting targets in the upper quadrants following 0,1, 2 or 3 consecutive successful detections (NL) in the same quadrant. Other conventions are the same as in Figure 4B. C. Task configuration for analyzing target detection performance in a lower quadrant as a function of the number of consecutive detections in the opposite quadrant. D. (Left) Change in the probability of detecting targets in the upper quadrants following 0, 1 or 2–3 consecutive successful detections (NU) in the opposite quadrant. Other conventions are the same as in Figure 4D. (PDF) [file pone.0064136.s009.pdf]
