# Supplementary material for: Spatial Probability Dynamically Modulates Visual Target Detection in Chickens
Source: PLoS One. 2013 May 29;8(5):e64136. doi: 10.1371/journal.pone.0064136 (PMC3667102; doi:10.1371/journal.pone.0064136)
Supplement: Figure S7 — Detection rates and reaction times in the HiP and LoP conditions for the lower hemifield locations. a. (Left) Contrast-response functions (population summary data) based on detection rates for lower hemifield targets for the HiP and LoP conditions. Other conventions are the same as in Figure 3C. (Right) Contrast-response functions (population summary data) based on reaction times for lower hemifield targets for the HiP and LoP conditions. Other conventions are the same as in Figure 3D. b. Same as figure S6A, but detection rates for lower hemifield targets for individual birds. c. Same as figure S6B, but reaction times for lower hemifield targets for individual birds. (PDF) [file pone.0064136.s007.pdf]

Figure S7

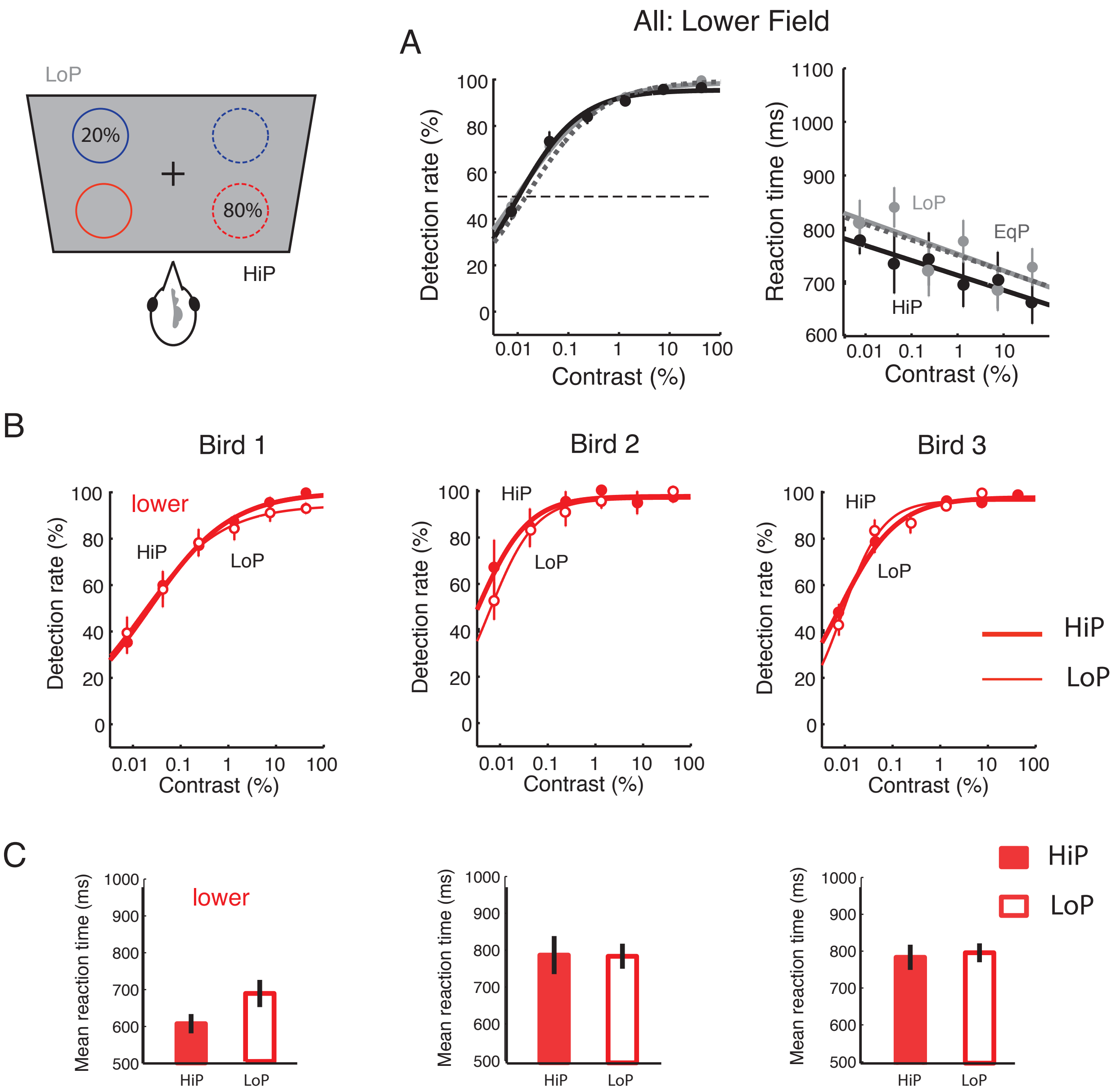

All: Lower Field
A
LoP
20%
80%
HiP
Detection rate (%)
Contrast (%)
Reaction time (ms)
LoP
EqP
HiP
B
Bird 1
Bird 2
Bird 3
lower
HiP
LoP
Detection rate (%)
Detection rate (%)
Detection rate (%)
Contrast (%)
C
lower
Mean reaction time (ms)
HiP
LoP
